# Disability, support and long-term social care of an elderly Spanish population, 2008-2009: an epidemiologic analysis

**DOI:** 10.1186/s12939-016-0498-2

**Published:** 2017-01-09

**Authors:** J. Almazán-Isla, M. Comín-Comín, E. Alcalde-Cabero, C. Ruiz, E. Franco, R. Magallón, J. Damián, J. de Pedro-Cuesta, L. A. Larrosa-Montañes, on behalf of the DISCAP-ARAGON Research Group, Javier Almazán, Fuencisla Avellanal, Enrique Alcalde, Juan Manuel Castellote, Javier Damián, Joao Forjaz, Belén Frades, Pablo Martínez-Martín, Jesús de Pedro Cuesta, Javier Virués-Ortega, Magdalena Comín, Ana Peña, Alarcos Cieza, Heinrich Gall, Geoffrey Reed, Olga Burzaco, Esther Franco, Cristina Ruiz, Cristina Martinez, Natividad Romanos, Maria José Tris, Susana Coloma, Rosa Magallón, Gloria Martín Gracia

**Affiliations:** 1 National Center for Epidemiology, Carlos III Institute of Health, Madrid, Spain; 2 Consortium for Biomedical Research in Neurodegenerative Diseases (Centro de Investigación Biomédica en Red sobre Enfermedades Neurodegenerativas - CIBERNED), Ministry of Science and Innovation, Madrid, Spain; 3 School of Health Sciences, University of Zaragoza, Zaragoza, Spain; 4 Department of Social Services and Family, Aragon Regional Authority, Zaragoza, Spain

**Keywords:** Disability, ICF, Prevalence, Functional dependence, WHODAS 2.0, Social services, Public health services

## Abstract

**Background:**

Though poorly known, relationships between disability, need of help (dependency) and use of social services are crucial aspects of public health. The objective of this study was to describe the links between disability, officially assessed dependency, and social service use by an industrial population, and identify areas of inequity.

**Methods:**

We took advantage of a door-to-door survey conducted in the Cinco Villas district, Spain, in 2008–2009, which provided data on disability, morbidity, and service use among 1216 residents aged ≥50 years, and officially assessed dependency under the 2006 Dependency Act (OAD). Using logistic regression, we combined data collected at homes/residences on 625 disability screened-positive participants, and administrative information on degree of OAD and benefits at date of visit.

**Results:**

Based on 163 disabled persons, the prevalence of residential/community-care users was 13.4% overall, with 6.0% being market-provided, 2.5% supported by the 2006 Act, and 4.9% supported by other public funds. Of 111 OAD applicants, 30 had been assigned an OAD degree; in 29 cases this was the highest OAD degree, with 12 receiving direct support for residential care and 17 receiving home care. Compared to unassessed dependency, the highest OAD degree was linked to residential care (OR and 95% CI) 12.13 (3.86–38.16), declared non-professional care 10.99 (1.28–94.53), and publicly-funded, non-professional care 26.30 (3.36–205.88). In contrast, 43 persons, 58% of the severely/extremely disabled, community-dwelling sample population, 81% of whom were homebound, including 10 persons with OAD but no implemented service plan, made no use of any service, and of these, 40% lacked a non-professional carer.

**Conclusions:**

Formal service use in the Cinco Villas district attained ratios observed for established welfare systems but the publicly-funded proportion was lower. The 2006 Act had a modest, albeit significant, impact on support for non-professional carers and residential care, coexisting with a high prevalence of non-use of social services by severely disabled persons.

## Background

The most powerful tool regularly used to describe how disability affects citizens and how the underlying social policies materialize, is the National Disability Survey. However, such surveys display limitations. Mulhorn and Threats, using the International Classification of Functioning, Disability and Health (ICF) [1] reported difficulties in comparing National Disability Survey figures based on measures of differing sensitivity [2]. A Spanish study showed that the 2008 Disability Survey afforded a data set of a size insufficient to be put in an ICF framework when it came to the Activity and Participation domains, and the authors stressed the lack of an individual disability score [3]. Notwithstanding this, disability measurement in national surveys is progressing [4]. The ICF disability instrument designed for population studies is the *World Health Organization* (WHO) *Disability Assessment Schedule-2.0* (WHODAS_2.0), used for screening (12 items) (WHODAS-12) and assessment (36 items) (WHODAS-36). The WHODAS-36 is expressly recommended by the WHO for epidemiologic surveys on disability [5], and its validity has been shown to be high [5–7].

Spain’s medium-sized, mixed, welfare state combines a number of models (Bismarckian, social democratic and social assistance) [8]. As a result, long-term (non-health) care (LTC) services for the aged have been heavily influenced by familialism and have traditionally been provided to the majority of the elderly on an informal basis and, only in the case of the most affluent strata, on a private, for-profit basis [9]. The 2006 Promotion of Personal Autonomy and Care of Dependent Persons Act (“Dependency Act”) (*Promoción de la Autonomía Personal y Atención a las personas en situación de dependencia*) [10], marked a turning point in national LTC policy. As a culmination of Europeanization processes dating back to the mid-1990s, which led to a convergence of welfare states in the European Union (EU) [11], the 2006 Dependency Law sought to ensure universal access to LTC services for residents on a decentralized basis, and rationalized this in terms of need and benefit categories [9]. Since 2007, official dependency assessments and individual support service plans (ISSPs) have been implemented countrywide under the above Act [10].

The aim of this study was to describe relationships between disability and LTC services, and to assess the coverage and potential impact of the 2006 Dependency Act on use of social services by disabled middle-aged and elderly residents in a rural-semirural population [12, 13].

## Methods

### Study population

Cinco Villas (population approximately 33,000 in 2008) is a district made up of 48 municipalities located in the Province of Zaragoza (northeastern Spain). This area was selected due to the logistic support provided by local authorities and non-governmental organizations, and because it constitutes the administrative unit for provision of social services. In 2008, the total population aged 50 years or over numbered 13,315 (Spanish National Statistics Institute). Health care was provided cost-free by five publicly-run primary-care centers, a university teaching hospital 85 km away in the city of Zaragoza, and more recently by a specialized, mainly out-patient, center located in Ejea de los Caballeros, the district’s main town. Sheltered accommodation was available at several homes for the elderly, which were either privately owned or operated by charities, and offered by a few municipalities. The study was conducted on 1360 *de facto* residents of Cinco Villas, drawn as a probabilistic sample from 12,784 social security card holders (age ≥50 years) [12]. As previously reported, after excluding 110 persons who declined to participate directly, the study was conducted on the overall participating proportion of the abovementioned 1360 residents, i.e., 1250 persons (91.9%). After excluding 34 individuals with incomplete data, the prevalence sample was made up of 1216 persons [12, 13]. Using a personal identifier, the same population was studied as follows: first directly, by undertaking a field survey from 2008 through 2009; and second, by registering linkage in 2010 to administrative social service data generated since 2007 pursuant to the 2006 Act.

### Assessments

#### Combined field survey of disability and services

Data were collected in two stages, screening and full assessment.

##### Screening

The WHODAS 2.0, a non disease-specific tool for assessment of disability, was deemed suitable, due to the considerably high number of diagnoses involved in epidemiologic and non-clinical studies. Data on socio-demographic characteristics (sex, age, marital status, living arrangements and education) and cognitive status were collected for the entire sample, and individuals were then screened using the WHODAS 12-item, a shortened version of WHODAS-36 [14]. The threshold for screening positive when using the 12-item version was a minimum of one positive answer. The *Mini-Examen Cognoscitivo* [15], the Spanish version of the Mini-Mental Status Examination, was used for assessing cognitive status. Subjects with a score <24 points (range 0 to 35) were also deemed to be positive to screening and underwent complete assessment.

##### Full assessment

Participants who screened positive for disability or cognition, 625, underwent assessment using a protocol focused on primary-care diagnoses, disability, lifestyle, and use of health and social resources. Information on diagnoses was obtained mainly from medical records in primary care, reports by health professionals and, in a few cases, proxy- or self-reports, creating a list of 26 prevalent and relevant chronic conditions in older people. Disability (see prevalence reported for Cinco Villas) [6, 13] in the Activity and Participation domains was evaluated with the WHODAS-36 [14], a questionnaire that assesses difficulties in six of these, i.e., understanding and communication, getting around, self-care, getting along with people, life activities, and participation in society. Items are answered on a 5-point Likert-type scale, ranging from 0 (no difficulty) to 4 (extreme difficulty). Global scores were calculated using the WHO Spanish Official Group scoring rules [6], and categorized as: 1-no problem (0–4%); 2-mild (5–24%); 3-moderate (25–49%); 4-severe (50–95%); and 5-extreme/complete problem (95–100%). We obtained global WHODAS-36 scores >4% for 604 of a total of 1214 persons with known age, and prevalence figures for mild, moderate, severe, and extreme disability, reported as 26.8, 16.0, 7.6 and 0.1%, respectively [13]. Given the low proportion of individuals presenting with extreme/complete WHODAS-36 disability, 0.1% in this study, the latter two categories were collapsed into one (4-severe and extreme/complete). Detailed data on WHODAS-36 disability prevalence and strong associations, e.g., with diagnoses obtained mainly from the five primary-care centers, Katz dependency, weekly carer hours, and homebound status, can be found elsewhere [13]. Additionally, we used a structured questionnaire to collect data during the disability survey visit, directly or from surrogate informants - mostly relatives and carers- on family and professional support services (home and personal care), and on residential care from institutions for sheltered accommodation, and the possible receipt of public funds for such support. The questionnaire sections on carers and use of social services were designed by taking into account the well-known, traditional role played by families in the care of elderly disabled, and the possible alternative outcomes of official dependency assessments in terms of support.

The disability-survey field work was conducted across the period, June 2008-June 2009. Four evaluators trained by members of the WHO Spanish Official Group (S. Herrera) and WHO ICF Reference Group (A Cieza, G Reed), made the survey visits to homes or institutions for assessment purposes [12, 13].

#### Register linkage study. Official dependency assessment and service plan

##### Long-term (non-health) care in Spain

The LTC system in Spain has been classified as being among those of familialist Mediterranean welfare states [16]. In a recent comparative analysis of the Spanish welfare system [17], the authors emphasized that, historically speaking, the major change in social-service policy was established at the end of 2006 by the promulgation of the Dependency Act [10]. This policy gave rise to the use of the Dependency Assessment Measure (*Baremo de Valoracion de la Dependencia*), which, to our knowledge, has never been validated [18]. This measure gives a 0-100 point score, which, once duly stratified, corresponds to the three degrees (DDs) and levels of dependency defined and shown in numbers in Table 1. When it comes to benefits, Sarasa [9] summarizes the position as follows: need of personal help has to be covered by four benefits in kind (institutional care, day-and-night centers, alarm-call service, and home-help services which include personal care and household help) and cash benefits, such as vouchers for services contracted in the market.

**Table 1:** Distribution of the positive-screened sample population according to WHODAS-36 global score in different strata, by degree of functional dependency as assessed using the official scale

Assessment status as per official functional dependency scale. Degree, level, and score shown in brackets	Number of persons in WHODAS-2 36 items and proportion [percentages] of screened sample				
	Low/no problem 0–4	Mild disability 5–24	Moderate disability 25–49	Severe/extreme disability 50–100	All scoreintervals 0–100
Degree I^a^ Both levels (25–49)	0	0	5	5	10
Degree II^b^ Lower level (50–64)	0	3	2	6	11
Degree II^b^ Higher level (65–74)	0	0	2	4	6
Degree III^c^ Lower level (75–89)	0	0	5	11	16
Degree III^c^ Higher level (90–100)	0	0	1	23	24
Assessed with degree assigned	0	3	15	49	67
Assessed without any degree assigned	0	3	1	3	7
All officially assessed	0 [0]	6 [2]	16 [8]	52 [56]	74 [12]
All not officially assessed	19 [100]	312 [98]	179 [92]	41 [44]	551 [88]
All assessed and unassessed	19	318	195	93	625

^a^Moderate functional dependency: needs personal help for basic ADL and personal autonomy on a limited, intermittent or once-per-day basis

^b^Severe functional dependency: needs support for several basic ADL several times per day but not for permanent, extensive personal care

^c^Extreme functional dependency: needs personal help or supervision for basic activities several times per day or continuously

Officially, disability levels were denoted as 1 and 2. Here notation changed to Lower and Higher, respectively, to avoid confusion between phonetically similar degrees and levels

Regional authorities are tasked with officially assessing need of help (dependency) and drawing up an ISSP. Where care cannot be provided through public social services, a related financial allowance is granted to cover the expected cost of the services envisaged under a private contract with carers or institutions of the applicants’ or relatives’ choice. Municipal social services providing home help or residential care have traditionally been supported by their own in-house budgets and, more recently, by regional funding allocated to ISSPs.

##### The National Dependency Care System data bank (NDCSDB)

Since 2007, the Autonomy and Dependency Care System (*Sistema para la Autonomía y Atención a la Dependencia*), a mixed state and regional body, coordinates public and private resource utilization for the care of dependent persons, and regularly reports statistics (http://www.imserso.es/imserso01/index.htm). The analysis of the practical effect of the 2006 Act on the study sample was conducted when data-collection for the disability survey had ended. In the latter part of 2009, a request was sent to the Social Services Authority to provide the results of all possible official assessments undergone by each of the 625 positively screened subjects [12]. Data on personally assigned DDs I, II or III and levels within each DD (lower and higher), as well as results of ISSP-approved services and those actually implemented before the survey visit, were obtained from the Regional Authority in 2010 and updated to July 2013 by the NDCSDB [12].

### Data-analysis

First, we cross-checked official dependency assessments for applicants before the visit date against WHODAS-36 global scores. Second, we performed crude analyses of differences in personal characteristics between users of LTC social services among the 93 persons shown to be severely/completely disabled by the WHODAS-36. Third, we described the content of ISSP rulings with respect to applicants’ official dependency degrees, which predated disability assessment by WHODAS-36 category in ignorance of the ISSP-ruling date. Fourth, from tables and, where possible, from logistic regression, we described DDs as predictors of different types of social services implemented at the visit date (including those entitled to ISSP), adjusting for WHODAS-36 disability, age, sex, town size, and living alone. We summarized these social services into three categories: residential services; home help (including day-care); and each of these when receiving publicly-funded support (PFS). Lastly, we assessed the impact of health conditions, registered at primary-care centers and grouped as reported elsewhere [13], on the use of different services and support. Etiologic fractions (EFs), i.e., the percent of different binary categories of service users corresponding to diagnostic entities were calculated using established formulae [19, 20]. When several DD categories were implicated, point estimates and 95% CIs for median EF values were obtained using bootstrap methods [21].

## Results

### Calendar time intervals for disability, service use and official dependency assessments

**Fig. 1:**
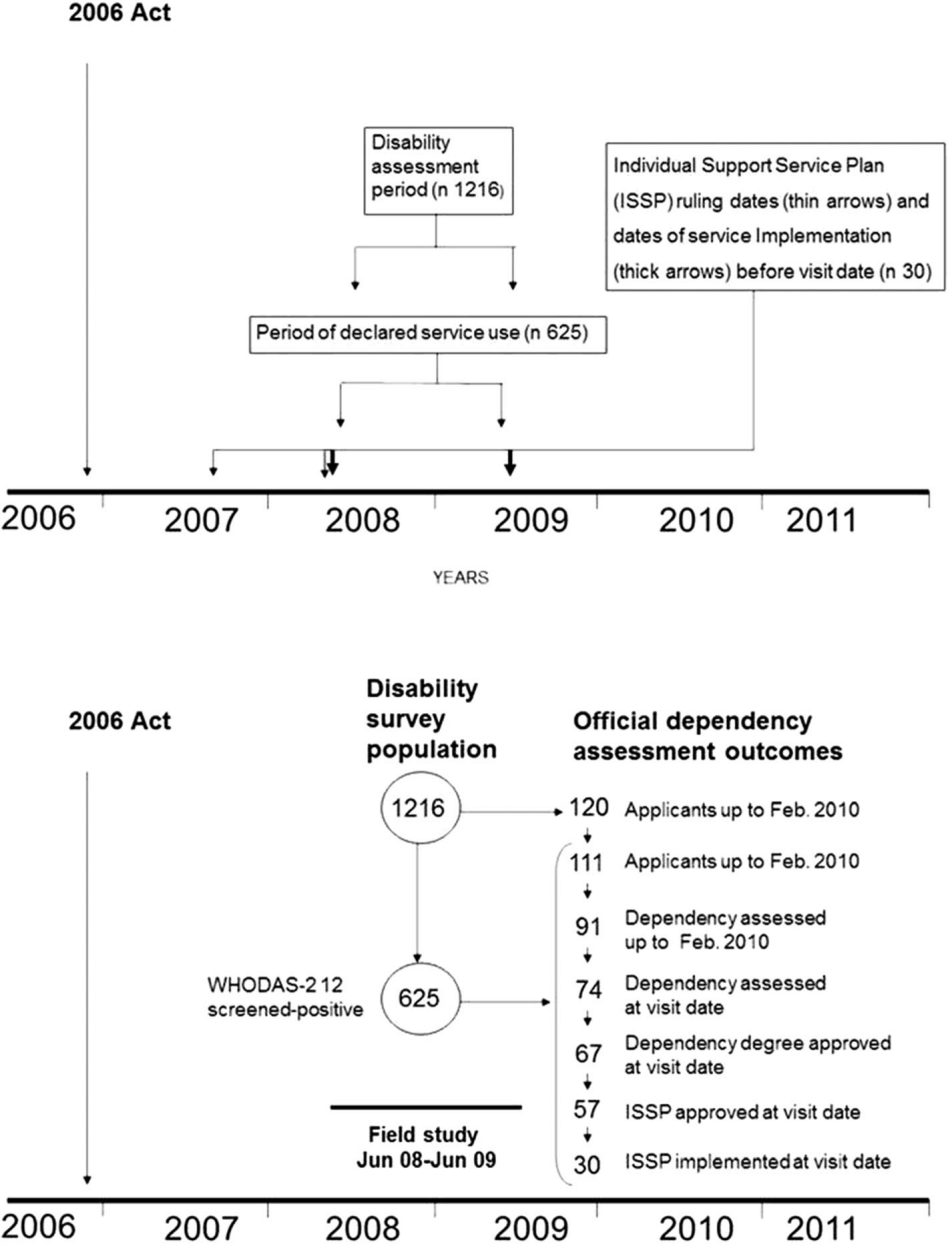
Time and study population. *Top*: time relationships between the entry into force of the 2006 Act, official dependency assessments, and service implementation following the 2006 Act (registered), as well as disability assessments and data on service use collected in the field survey. *Bottom*: attrition flow for 111 disabled and non-disabled study participants to 30 users of social services, linked to implementation of the 2006 Act prior to visit

A summary of the time relationships between disability, service-use assessment, official dependency assessment, and service-plan assignment is depicted in Fig. 1. By the end of February 2010, ISSPs were issued for 57 of 625 screened persons, with these plans being implemented in 30 cases before the visit date. The duration of the interval between the date of application to that of implementation for these 30 persons, 29 with degree-III disability (DD-III) and one with DD-II, was a mean of 358.5, SD 97.9 days. To sum up, early statutory service implementation under the 2006 Act was considerably delayed and restricted to the most severely disabled.

### The view of disability vs. officially assessed dependency at visit date

Table 1 shows the moderate correspondence between disability and dependency as seen from distributions of the 625 screened positive subjects, with a breakdown by WHODAS-36 categories (columns) and official dependency assessment results. Of 93 persons rated severely/completely disabled by WHODAS-36, fifty-two, 56%, had been officially assessed, and forty-nine, 53%, had been assigned DD I (5), DD II (10) and DD-III (34), namely, moderate, severe and extreme functional dependency, respectively. Six, 15%, of 40 participants with highest dependency, DD-III, were classified as moderately disabled. In summary, at the visit date the prevalence of highest global WHODAS-36 disability outnumbered that of severe and extreme OAD by 63%.

### Social services approved and implemented by disability and dependency groups under the 2006 Act

ISSPs, mutually exclusive service-packages, either ever-approved or enforced, are shown in Table 2, broken down by DD, WHODAS 2.0 category, and age. Of the above 625 screened positive subjects, fifty-seven, 9%, were entitled to support (21 for residential care, 35 for home care, and 1 for care at a day-center); and of these 57 persons, 30, all but one being DD-III, received ISSP services: 12 for residential care; 17 for home help; and one, DD-II, for day-care. Benefit entitlements under the 2006 Act had been implemented, as planned, for only 29 of 30 severely or extremely dependent persons; 25, 83%, were rated as severely-extremely disabled by WHODAS-36 scores; 21, 70%, were women (not shown in Table 2); and 24, 80%, were aged ≥80 years. Up to the date of the visit, the 2006 Act had covered 2.5% of the study population, with this encompassing services implemented at both community (17/1216, 1.4%) and residential levels (13/1216, 1.1%).

**Table 2:** Number of persons with ISSP-approved and -implemented (in brackets) social services, broken down by disability, dependency degree (DD), and age

	Service users as a result of implementation of the 2006 Act									
WHODAS-36 score or age-group	Dependency Degree II beneficiaries			Dependency Degree III beneficiaries		Overall service beneficiaries	Prevalence of ISPP beneficiaries in populations [%]			
	Financial support for professional or non-professional home care	Financial support for residential care	Financial support for day care center	Financial support for professional or non-professional home care	Financial support for residential care		Among population screened positive for disability		Among population aged >50 years including persons screened negative for disability	
							Approved	Implemented	Approved	Implemented
0–4	-	-	-	-	-	-	0/19 [0]	0 [0]	0/610 [0]	0/610 [0]
5–24	3 (0)	-	-	-	-	3	3/318 [1]	0 [0]	3/318 [1]	0/318 [0]
25–49	-	4 (1)	-	1 (0)	5 (3)	10 (4)	10/195 [5]	4/195 [2]	10/195 [5]	4/195 [1]
50–95	7 (0)	2 (0)	1 (0)	23 (16)	10 (9)	43 (25)	43/92 [47]	25/92 [27]	43/92 [47]	25/92 [11]
96–100	-	-	-	1 (1)	-	1 (1)	1/1 [100]	1/1 [100]	1/1 [100]	1/1 [100]
Total	10 (0)	6 (1)	1 (0)	25 (17)	15 (12)	57 (30)	57/625 [9]	30/625 [5]	57/1216 [5]	30/1216 [2]
							Population and prevalence			
50–59y	-	-	1	-	1 (1)	2 (1)	1/101 [2]	1/299 [1]		
60–69y	4	1	-	-	-	5 (0)	0/116 [0]	0/305 [0]		
70–79y	1	1	-	6 (4)	2 (1)	10 (5)	5/198 [6]	5/352 [3]		
80–89y	2	3	-	11 (8)	10 (9)	26 (17)	17/174 [19]	17/221 [14]		
≥90y	3	1 (1)	-	8 (5)	2 (1)	14 (7)	7/34 [42]	7/37 [37]		
All	10 (0)	5 (1)	1 (0)	25 (17)	15 (12)	57 (30)	30/623 [9]	30/1214 [5]		

Percentage of users among the screened positive disabled, and total study population in square brackets

Ten persons with DD I were assigned no services

*ISSP* Individual support service plan

### Overall service use and distribution by relevant groups

The global descriptive view of LTC services is seen here from different perspectives, obtained from disaggregated data shown in Table 3 or otherwise from tabulations explicitly mentioned in the text.

**Table 3:** Number of long-term care (LTC) services, broken down by disability, dependency degree and age

	Number of LTC service users							
WHODAS-36 score or age group	Study participants using services unrelated to officially assessed dependency (in brackets those with publicly-funded support (PFS))			Dependency Degree II+ III beneficiaries		All users	Prevalence of PFS service users [%]	
	Declared professional or non-professional home help for home care	Residential care	Day care center (DCC)	Professional or non-professional home care	Residential care		Among population screened positive for disability	Among surveyed population including persons screened negative for disability
0–4	-	1 (0)	-	-	-	1	0/19 [0]	0/610 [0]
5–24	38 (12)	12 (6)	5 (0)	-	-	55 (18)	18/318 [6]	18/318 [6]
25–49	36 (21)	15 (8)	3 (2)	-	(4)	58 (35)	35/195 [18]	35/195 [18]
50–95	12 (8)	9 (2)	2 (1)	(16)	(9)	48 (36)	36/92 [39]	36/92 [39]
96–100	-	-	-	(1)	-	1 (1)	1/1 [100]	1/1 [100]
Total	86 (41)	37 (16)	10 (3)	(17)	(13)	163 (90)	90/625 [14]	90/1216 [7]
							Number of users, population, and prevalence	
50–59y	7 (2)	2 (1)	-	-	(1)	10 (4)	4/101 [10]	4/299 [3]
60–69y	7 (1)	2 (2)	-	-	-	9 (3)	3/106 [6]	3/305 [2]
70–79y	24 (10)	11 (5)	5 (1)	(4)	(1)	45 (21)	21/198 [22]	21/352 [12]
80–89y	43 (23)	17 (6)	5 (2)	(8)	(9)	82 (48)	48/174 [57]	48/221 [44]
≥90y	5 (5)	3 (1)	-	(5)	(2)	15 (13)	13/34 [66]	13/37 [60]
All	86 (41)	35 (15)	10 (3)	(17)	(13)	161 (89)	89/623 [29]	89/1214 [14]

Persons receiving individual support service plan (ISSP)-linked services (in brackets). Prevalence of publicly-fund-supported (PFS) service users

#### Crude prevalence of LTC users by service category and funding sources

The prevalence of LTC-service users was 163/1216, 13.4%, with 4.1%, 50, being users of residential services, and 9.3%, 113, being users of community services (home help and day care). PFS-based LTC services were reportedly received by 90/1216, 7.4% of the study population, with 30 of these persons benefiting from ISSP assessments: the 90 users generated a 5.0% prevalence of community care users, with 61 users, and a PFS-based sheltered housing prevalence of 2.4%, with 29 users of residential services. The prevalence of overall market-based (non-PFS) social service use (calculated from Table 3) was 6.0%, 73 persons, with 4.3%, 52 persons, being users of residential care and 1.7%, 21 persons, being users of community services. The bulk of such services (60 of 73 users, 82%) was received by the mild and moderately disabled, as opposed to the severely disabled. In other words, use of PFS-based services was higher than that of market-based services, particularly for residential living. The 2006 Act covered 28% of PFS-based community services and 55% of PFS-based residential services. To recapitulate, the overall prevalence of disability-related LTC use was 13.4%, 6.0% market-based and 7.4% public fund-based; of the latter, 2.5% was under the 2006 Act and 4.9% was supported by other public funds. Community services predominated among services provided by the market.

#### Patterns of service use by age and disability

Overall use of LTC services increased with age. Combining data for ages ≥80 years, disaggregated in Table 3 (left block for users, right for denominators), the proportion of LTC service users was 36%, 97 out of 258: 24% was PFS-based, 7% for residential care and 41/258, 16%, for home help. Among the population aged 65 years and over, total social service use, a frequently reported indicator not available from the figures in Table 3, was 5.4%, with PFS-based residential and community care being 2.0% and 3.5%, respectively.

**Fig. 2:**
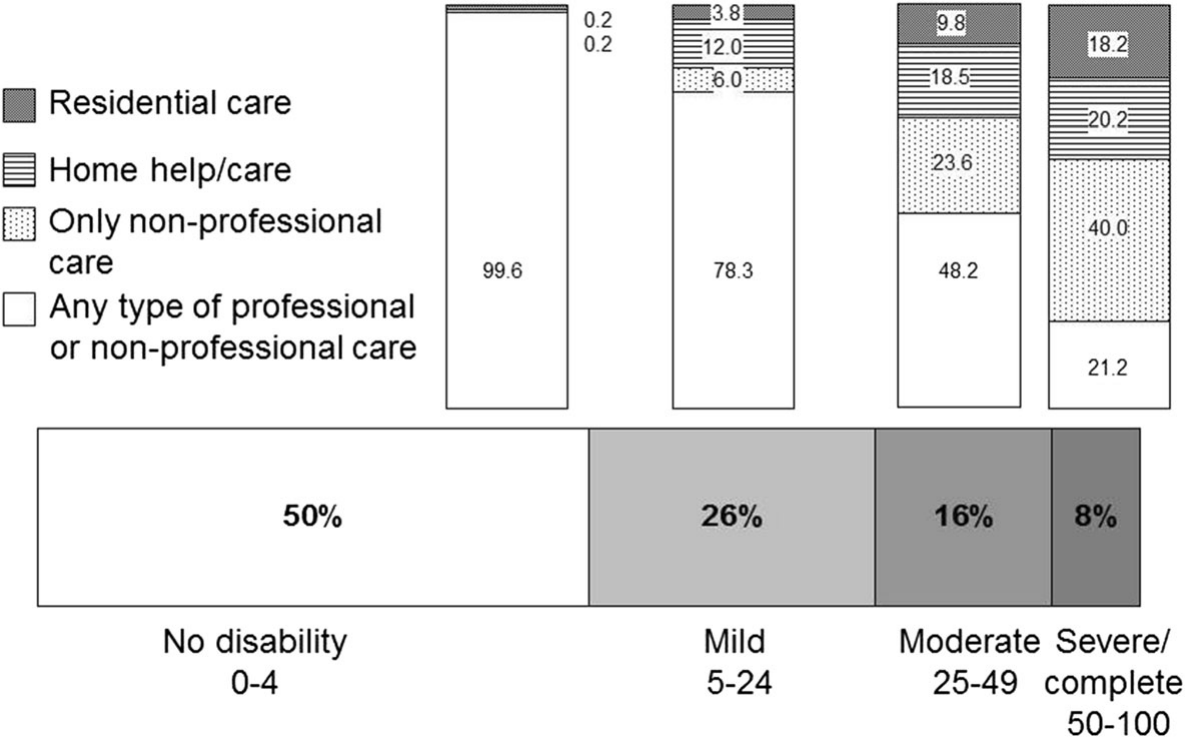
Disability-related service use and support received by participants according to the Cinco Villas survey. One person screened positive with WHODAS-36 score 0-4 in sheltered housing, is not depicted

Table 3 shows the distribution of LTC services and support for all users by disability level: the majority of users of PFS-based community services, 72 out of 90, had moderate or severe/extreme WHODAS-36 disability, and (shown at right) the prevalence of PFS-based service users among the disability-score stratified sample increased from 0 to 100%. The changing panorama of service use for disability groups depicted in Fig. 2, reveals that a high proportion of severely/extremely disabled subjects, 40%, received non-professional care only, and 21% lacked any kind of service or support.

### The analytical perspective of social service use

Predictors of official dependency assessment, as well as use of social services in different formats (declared, declared with PFS, and declared or implemented under an ISSP), are shown in Table 4. Dependency assessment, contact with social care office, declared or registered home help, and professional or non-professional care -whether supported or not supported by public funding- were associated with age (per year, data not shown) and with WHODAS-36 scores (per point). Residence in towns >500 inhabitants (not shown in Table) was linked to an increased frequency of declared PFS home help for personal or domestic tasks (OR and 95% CI) 4.07 (2.34–7.08). These figures for persons living alone were 6.97 (2.92–16.66).

**Table 4:** Variation in service use, including financial support, as a result of official dependency assessments or other sources, obtained from logistic models

Service or service-linked resource used	Dependency not assessed^a^ -Users -Non-users	Dependency assessed -Users -Non-users	No. of records in models^a^	WHODAS-36 per point	DD I ref. unassessed	DD II ref. unassessed	DD-III ref. unassessed	Etiologic fraction with 95% CI
Contact with Social Care Office	57 498	14 53	619	1.03 (1.01–1.04)	0.72 (0.08–6.31)	5.80 (1.92–17.52)	0.76 (0.23–2.51)	5.77 (-11.92–18.39)
Official dependency assessment	7 (without DD) 551	67	625	1.08 (1.06–1.09)	-	-	-	-
Residential living	34 524	23 44	622	0.99 (0.97–1.01)	1.90 (0.22–16.54)	8.60 (2.63–28.07)	12.13 (3.86–38.16)	36.19 (20.35–50.97)
Residential living with PFS (declared)	13 544	13 54	612	0.99 (0.96–1.01)	1.00 (1.00–1.00)	12.15 (2.56–57.77)	25.50 (5.35–121.61)	47.49 (26.50–68.75)
Residential living supported by 2006 Act (registered)^b^	0 595	13 17	521	0.96 (-0.91–1.02)	-	-	-	-
Residential living with PFS (declared or under 2006 Act)^b^	16 579	13 17	621	0.99 (-0.96–1.02)	-	-	41.18 (8.40–201.84)	39.40 (20.33–58.13)
Home help for domestic tasks (declared)	58 466	8 36	567	1.01 (0.99–1.03)	1.51 (0.17–13.81)	1.42 (0.25–8.02)	1.25 (0.31–5.05)	3.2 (-10.62–14.15)
Home help for personal care (declared)	15 509	8 37	567	1.03 (1.00–1.05)	3.19 (0.32–32.14)	9.62 (1.91–48.50)	2.07 (0.36–11.96)	24.11 (-15.74–49.48)
Home help for personal care or domestic tasks with PFS (declared)	33 491	7 37	567	1.03 (1.00–1.05)	2.58 (0.25–26.40)	2.23 (0.35–14.27)	0.85 (0.16–4.41)	3.52 (-35.4–20.97)
Available non-professional carer (declared)	86 438	36 71	567	1.04 (1.02–1.06)	4.47 (0.93–21.50)	7.50 (1.59–35.32)	10.99 (1.28–94.53)	19.4 (2.31–33.73)
Non-professional carer with PFS (declared)	2 522	15 28	471	1.05 (1.01–1.09)	-	36.57 (4.74–281.96)	26.30 (3.36–205.88)	83.16 (-0.61–94.97)
Professional carer (declared)	41 483	9 34	558	1.02 (1.00–1.04)	-	2.56 (0.58–11.31)	0.97 (0.26–3.61)	2.66 (-36.1–21.21)
Carer (declared)	115 409	37 6	567	1.04 (1.03–1.06)	2.81 (0.58–13.57)	8.47 (1.55–46.16)	6.35 (0.75–54.05)	12.96 (-0.57–26.15)
Carer with PFS (declared)	22 502	20 23	558	1.04 (1.02–1.06)	1.67 (0.16–16.95)	7.83 (1.58–38.77)	6.41 (1.73–23.70)	39.85 (18.00–59.13)
Carer supported under 2006 Act^b^	0 524	16 ^c^ 27	567	1.05 (1.02–1.07)	-	-	-	-
Carer with PFS (declared or supported under 2006 Act)^b^	22 502	34 1	567	2.13 (-1.15–3.93)	-	-	1.13 (-1.06–1.20)	4.06 (0.71–8.16)

Adjusted for age, sex, WHODAS-36 score, living alone, municipality size, OR and 95%CI. Dependency degree (DD). Publicly-funded support (PFS)

^a^Including number of assessed individuals without assigned DD

^b^Calculated using individual support service plan (ISSP) date of effective benefit

Additionally, eight of 522 persons used day-care centers and 139 of 544 persons had household adaptation

Relevant features of LTC service use partly reflecting the impact of the 2006 Act are illustrated by the highest ORs for DD-III in: PFS residential living, OR 95% CI, 41.18 (8.40–201.84), accounting for an etiologic fraction (ET) of almost 40%; PFS non-professional carer, 26.30 (3.36–205.88); and PFS carer, 6.41 (1.73–23.70). High ORs seen for the DD-II group (not covered by the 2006 Act, which supported 29 DD-III and one DD-II users) were for declared home-help for personal care, 9.62 (1.91–48.50), and for PFS non-professional care, 36.57 (4.74–281.96). To sum up, the results in Table 4 show that a large majority of LTC service users, whether with or without PFS, were participants whose dependency had not been officially assessed.

### Equity among the severely/extremely disabled

A comparative view of the personal, sociodemographic and residential characteristics of the 74 prevalent, severely/extremely disabled community-dwelling study participants, broken down by use vs. non-use of at least one service (disregarding the official disability assessment) is shown in Table 5. In addition to a 3-year older mean age, relevant differences between the 31 users and 43 non-users were, in the case of non-users: higher proportions diagnosed with dementia, 45% vs. 16%, and stroke, 58% vs. 12%; and a higher proportion receiving support from non-professional carers, 24/31, 77%, vs. 17/43, 40%. The proportions of history of depression were quite similar, with the two groups registering similar proportions, with EURO-D scores ≥4, 52% vs. 47%, respectively [22, 23]. No clear patterns were in evidence, whether by educational level and population size, or by administrative status of residential nucleus (data not shown). When the whole severely/completely disabled group of 93 was considered (as seen in Tables 1, 3 and 5), the service non-user group, encompassed approximately half of the severely/completely disabled population, i.e., 43 of the 93, and was 81.40% homebound, i.e., 35 persons. In sum, approximately, one in four resided in an institution, one had a home-based professional carer, and two in four received no service whatsoever. Six, 6.5%, were living alone.

**Table 5:** Characteristics of study participants classified as severely/extremely disabled by the WHODAS-36, living at home, and grouped by service-user status, i.e., users of at least one social service and social service non-users

Personal, socio-demographic, clinical and residential features	Used at least one service: home help, day care, professional carer or had an individual support service plan (ISSP) implemented	Social service benefit non-users	*P*-values for differences of proportions or means
Number of individuals (*n* = 74)	31 (100)	43 (100)	
Social and demographic features			
Gender % (female)	22 (70.97)	34 (79.07)	0.423
Age in years. Mean (SD),	82.6 (9.02)	79.34 (10.80)	0.035
Academic qualification. None, incomplete primary, %	12 (38.71)	22 (51.16)	0.289
“*Can hardly make ends meet*”^a^	7 (23.33)	13 (30.90)	
Living alone^b^	2 (6.67)	4 (9.30)	0.657
Average number of household members. Mean (SD),	2.9 (1.32)	2.7 (1.04)	0.291
Available non-professional carer	24 (77.42)	26 (60.47)	0.121
Available professional carer	15 (48.39)	0 (0)	<0.001
Contact with social services unit (denoted as *Centro Base*)	7 (22.58)	4 (9.30)	0.1132
Diagnoses registered in primary care medical records^b^			
History of depression	9 (29.03)	10 (23.26)	0.575
Dementia	14 (45.16)	7 (16.28)	0.108
Chronic obstructive pulmonary disease	4 (12.9)	4 (9.30)	0.623
Urinary incontinence	6 (19.35)	3 (6.98)	0.108
Stroke	18 (58.06)	5 (11.63)	<0.001
Neurodegenerative disease	3 (9.68)	1 (2.33)	0.168
Average number of chronic conditions. Mean (SD)	3.45 (1.41)	2.90 (1.53)	0.140
Mini-Mental Status Examination score <24 at survey date^c^	11 (57.81)	24 (60.00)	0.084
Prevalence of depressive symptoms EURO-D score ≥4^d^	16 (47.1)	16 (51.6)	0.714
WHODAS-36 score			
Mean (SD)	70.25 (15.58)	61.54 (10.33)	0.005
Severe/extreme difficulties in “*Getting out of home*”^e^	27 (87.10)	35 (81.40)	0.512
Municipality size			
<500 inhabitants	13 (41.91)	16 (37.21)	0.681
500-14000 inhabitants	12 (38.70)	24 (55.81)	0.146
>14000 inhabitants	6 (19.35)	13 (6.98)	0.108
Individual support service plan implemented	17 (54.83)	0 (0)	<0.001
Individual support service plan not implemented.	4 (12.90)	10 (23.26)	0.262

Percentages within each group in brackets

^a^Calculated with 30 and 42 persons for each group; ^b^Calculated with 30 and 43 persons for each group. ^c^Calculated with 19 and 40 persons for each group

^d^Calculated with 15 and 37 persons for each group. ^e^ Item D2.4: In the last 30 days, how much difficulty did you have in “Getting out of your home”?

### Service use by persons grouped in diagnostic categories

As shown in Table 6, the highest probability of use of services, as perceived from diagnosis-OR and diagnosis-EF with a lower 95%CI limit of over 1, was seen: 1) for residential care for persons who suffered from severe mental disease, OR 21.74, EF 11.19%, and hip fracture, OR 7.49, EF 5.86% (the latter not shown); 2) for ISSP-supported residential care, where the figures changed to dementia OR 12.52, EF 49.68%. Declared non-professional care was mostly linked to dementia, OR 11.1, EF 17.52%, and stroke, OR 5.42, EF 21.31%. In the case of professional carers, stroke, OR 5.42, EF 27.06%, ranked first. In brief, persons with dementia and vascular disease proved to be particularly relevant service group users.

**Table 6:** Results of logistic regression, i.e., OR and (95%CI) and etiologic fractions with (95%CI), the latter representing the percentage of total service use for selected diagnostic categories

Service used	Dementia	Severe mental disease	Cerebrovascular disease	Neurodegenerative diseases
Residential living	3.70 (1.62–8.44) 16.74 (3.56–29.96)	21.74 (4.71–100.24) 11.19 (0.00–20.91)	1.37 (0.59–3.15) 5.01 (-11.67–17.98)	4.31 (1.41–13.16) 9.04 (0.88–19.54)
Residential care supported by the 2006 Act.	12.52 (3.58–43.82) 49.68 (14.71–79.27)	8.23 (0.96–70.31) 12.54 (0–37.74)	1.35 (0.31–5.93) 6.40 (-43.69–43.71)	3.60 (0.45–28.63) 9.46 (-2.86–32.81)
Household adaptation	1.56 (0.70–3.46) 2.50 (-2.39–7.25)	-	1.57 (0.90–2.76) 5.46 (-2.18–13.32)	1.59 (0.51–5.02) 1.11 (-1.59–4.82)
Home help (declared)	0.63 (0.18–2.26) -2.37 (-7.76–4.00)	-	2.60 (1.30–5.21) 11.82 (2.02–22.73)	2.22 (0.58–8.43) 2.16 (-1.19–8.28)
Available carer (declared, any type of carer)	7.95 (3.32–19.03) 13.98 (7.67–20.10)	25.38 (0.41–71.34) 0.10 (-0.09–2.97)	4.81 (2.69–8.60) 19.30 (11.82–27.03)	4.68 (1.52–14.42) 3.82 (0.27–7.93)
Non-professional carer (declared)	11.11 (4.61–26.81) 17.52 (10.27–24.47)	7.84 (0.56–109.73) 0.33 (0.00–3.40)	4.78 (2.63–8.68) 21.31 (11.46–29.93)	3.71 (1.16–11.88) 3.53 (-0.02–7.42)
Professional carer (declared)	0.93 (0.27–3.25) -0.97 (-22.04–11.85)	-	5.42 (2.69–10.94) 27.06 (12.45–41.63)	3.62 (0.92–14.23) 4.11 (-0.20–11.96)
Personal care supported by the 2006 Act	48.06 (12.88–179.29) 63.36 (30.20–80.77)	-	12.70 (3.53–45.65) 54.19 (18.64–74.21)	9.19 (0.84–100.95) 5.24 (-6.75–15.89)

Adjusted for age, sex, and other diagnoses

Less relevant conditions in terms of category of service used (diabetes, depression, heart failure, chronic liver diseases, hip fracture, visual loss and peripheral arterial disease, among other) are not shown

## Discussion

To our knowledge, this is the first combined door-to-door disability screening and policy survey worldwide. The study shows a mixed public and private system that is transitioning towards a higher use of community as opposed to residential services, high overall service use, and reflects the impact of the 2006 Act’s implementation on residential services and PFS-based non-professional care. Two key features appear to be present: a high proportion of overall social service use supported by private funds and concentrated among the mildly and moderately disabled; and the presence of a large group of severely disabled non-users. Since sample selection was random, participation was high and the use of surrogate informants (generally carers) unavoidable, we feel that related limitations had only a modest impact on results.

Accrual of PFS-based community and residential services ranked midway on a European scale, in terms of both the magnitude of overall use and the ratio of community to residential services [24]. On October 1^st^, 2012, 38% of Sweden’s population aged >80 years received special forms of housing or were granted home help services in ordinary housing. This percentage was 58% higher than that in Cinco Villas in 2008–2009, where the proportion of persons aged ≥80 years benefiting from PFS-based services was 24% and total service use, 34%, was similar [25]. These results would suggest that the proportion of overall service users among the elderly is as high as that of established systems, though with lower use of PFS-based services and with the difference offset by market-based services and a considerable proportion of non-professional carers.

Dependence on another person’s help was only officially measured as a defined DD. However, dependency remained elusive for persons without a DD, namely, for the majority of the moderately or severely disabled study population. Since only DD-III participants benefited from the 2006 Act in practice, the high PFS-based service use by DD-I and particularly by DD-II participants must have been determined by support from sources other than the 2006 Act. We believe that adjustment for disability revealed existing PFS-based services for dependent participants who had been officially assessed but not yet given services, and the DD-II group in particular. The use by the D-II group of PFS-based services, such as residential living, help at home for personal care, and professional or non-professional carers, whether or not publicly supported–to a degree that was frequently as high as the use made of these same services by the DD-III group- would indicate the underlying, ubiquitous presence of PFS-based services provided across all DD groups by non-regional authorities. Such public institutions would most likely correspond to the largest municipalities which had well-established social service units in the Cinco Villas district in 2003. Stroke, dementia, and chronic heart failure might constitute ailments particularly suited to home-care, including that given by professional carers, entitled to support under the 2006 Act.

### The service non-user group at risk

We estimate that in Cinco Villas the proportion of severely disabled persons deprived of LTC services might have risen to over 400, i.e., 1.2% of the total population of 33,000, and approximately half a million of the estimated one million countrywide [3]. Lack of official assessment must be attributed to patients or carers not seeking official assessment. Failure to apply for official service entitlement cannot, however, be interpreted as low use of either market- or PFS-based social services. The study provides no clues as to why half of the severely disabled population did not use social services, whether or not ISSP-implemented, and why 21% lacked professional or non-professional home-based care. A lower frequency of disabling conditions such as dementia or some other comorbidity, diagnosed or undiagnosed, may explain part of this difference. In a previous study, diagnosed depression was strongly associated with disability [26]. In Cinco Villas, depressive symptoms, which in our case were similar in both groups, were associated with history of depression and highly disabling conditions, such as chronic heart failure [27]. In accordance with the abovementioned reports [28–32] and the proportion of undiagnosed cases seen in reviewed dementia surveys [33, 34], one could speculate that undiagnosed dementia among the population with Mini-Mental Status Examination scores <24 and a lower disability level with similar counts for depression, leads a proportion of the severely disabled elderly or their carers to refrain from seeking state or municipal service support. Determinants of such a phenomenon may have acted in Cinco Villas. The high mortality among disabled elderly [35], and the rapid transitions to more severe disability or death, i.e., one third after 30 months [36], suggest that the background dynamics of neglect in using social services correspond to a short interval in the life course, thus determining a high turnover which, in turn, would require undelayed action at an individual level.

The causes of unmet need of services are diverse and not always attributable to a lack of available resources. Resource allocation and service development traditionally focus on diagnosis and perceived needs [37]. Results from three studies on community patients with dementia highlighted the presence of: 1) high levels of unmet need in Ireland, particularly among patients with agitation and low social interaction [37]; 2) no or low use of support services by one in three, and one in four members, respectively, of a sample of dementia carers in Australia [28]; and, 3) at least one unmet need of care in virtually all participants who screened positive for dementia assessed in-home in Baltimore [31]. In two studies, perceived lack of need and lack of knowledge of services, though not service availability, were identified as major impediments to service use [28, 31]. Over the last decade, studies adopting different approaches have shown that a proportion of the disabled elderly enjoy limited access to services [30, 38]. Barriers to service use among the elderly have been identified and variously attributed to administrative oversight of patient disability by physicians [29], poor psychological adjustment to the experience of disability [39], denial of disability [32], and elderly self-neglect [40]. The abovementioned evidence supports the fact that non-use of services, including those made available by the 2006 Act in Cinco Villas, should be viewed as an important, widespread, disability-related, public health problem.

A unique strength of the study is its three-pronged population-based, disability- and services-oriented approach. A potential study weakness is the unknown validity of declared data and unstable effect measures, due to scarce data, particularly for diagnostic groups.

The main implications of the results of this study for the international community and the industrial world derive from the high recorded prevalence of severe-extreme disability, and the fact that approximately half of this group has not come into contact with statutory service providers. Such isolation might be a component of the reported low *social participation* in Cinco Villas [13]. Research into non-use of social services by severely disabled persons is called for. Since proposed areas for program development include strengthening referral networks between providers of medical and social services [41], social planning should perhaps incorporate some form of surveillance of unattended severe disability in primary care.

## Conclusions

To sum up, this study gives a population-based overview of social service use by the disability screened population aged ≥50 years in the Cinco Villas district. Our results identify and describe a non-user group accounting for half of the severely disabled, coexisting with a high prevalence of market-based or publicly supported residential- and home-care users. At the onset of the economic crisis in southern Europe, the 2006 Act had a modest yet significant impact on the consolidation of non-professional and residential care. Active primary-care surveillance of potentially unattended severe disability might be useful in identifying priorities and reducing the unmet need of services and support in some EU Member States.

## Abbreviations

ADL: Activities of daily living
DD: Dependency degree
EF: Etiologic fraction
EU: European Union
ICF: International Classification of Functioning, Disability and Health
ISSP: Individual Support Service Plan
LTC: Long-term care
NDCSDB: National Dependency Care System data bank
OAD: Officially assessed dependency
PFS: Publicly-funded support
WHODAS-12: WHODAS-2.0 for screening (12 items)
WHODAS-2.0: World Health Organization (WHO) Disability Assessment Schedule-2.0
WHODAS-36: WHODAS-2.0 for and assessment (36 items)
