# Erratum to: Disability, support and long-term social care of an elderly Spanish population, 2008–2009: an epidemiologic analysis

**DOI:** 10.1186/s12939-017-0562-6

**Published:** 2017-05-31

**Authors:** J. Almazán-Isla, M. Comín-Comín, E. Alcalde-Cabero, C. Ruiz, E. Franco, R. Magallón, J. Damián, J. de Pedro-Cuesta, L. A. Larrosa-Montañes, on behalf of the DISCAP-ARAGON Research Group

**Affiliations:** 1 National Center for Epidemiology, Carlos III Institute of Health, Madrid, Spain; 2 Consortium for Biomedical Research in Neurodegenerative Diseases (Centro de Investigación Biomédica en Red sobre Enfermedades Neurodegenerativas - CIBERNED), Ministry of Science and Innovation, Madrid, Spain; 3 School of Health Sciences, University of Zaragoza, Zaragoza, Spain; 4 Department of Social Services and Family, Aragon Regional Authority, Zaragoza, Spain

## Erratum

After publication of this article [1], it was noticed that Table 2 contained misalignment of figures. The corrected Table 2, below, contains the same figures as the original, with the numbers shown at bottom right properly aligned. The original article has also been updated to reflect this.

**Table 2:** Number of persons with ISSP-approved and -implemented (in brackets) social services, broken down by disability, dependency degree (DD), and age

	Service users as a result of implementation of the 2006 Act									
WHODAS-36 score or age-group	Dependency Degree II beneficiaries			Dependency Degree III beneficiaries		Overall service beneficiaries	Prevalence of ISPP beneficiaries in populations [%]			
	Financial support for professional or non-professional home care	Financial support for residential care	Financial support for day care center	Financial support for professional or non-professional home care	Financial support for residential care		Among population screened positive for disability		Among population aged >50 years including persons screened negative for disability	
							Approved	Implemented	Approved	Implemented
0-4	-	-	-	-	-	-	0/19 [0]	0 [0]	0/610 [0]	0/610 [0]
5-24	3 (0)	-	-	-	-	3	3/318 [1]	0 [0]	3/318 [1]	0/318 [0]
25-49	-	4 (1)	-	1 (0)	5(3)	10 (4)	10/195 [5]	4/195 [2]	10/195 [5]	4/195 [1]
50-95	7 (0)	2 (0)	1 (0)	23 (16)	10 (9)	43 (25)	43/92 [47]	25/92 [27]	43/92 [47]	25/92 [11]
96-100	-	-	-	1 (1)	-	1 (1)	1/1 [100]	1/1 [100]	1/1 [100]	1/1 [100]
Total	10 (0)	6 (1)	1 (0)	25 (17)	15 (12)	57 (30)	57/625 [9]	30/625 [5]	57/1216 [5]	30/1216 [2]
							Population and prevalence			
50-59y	-	-	1	-	1 (1)	2 (1)	-	1/101 [2]	-	1/299 [1]
60-69y	4	1	-	-	-	5 (0)	-	0/116 [0]	-	0/305 [0]
70-79y	1	1	-	6 (4)	2 (1)	10 (5)	-	5/198 [6]	-	5/352 [3]
80-89y	2	3	-	11 (8)	10 (9)	26 (17)	-	17/174 [19]	-	17/221 [14]
≥90y	3	1(1)	-	8 (5)	2 (1)	14 (7)	-	7/34 [42]	-	7/37 [37]
All	10 (0)	5(1)	1 (0)	25 (17)	15 (12)	57 (30)	-	30/623 [9]	-	30/1214 [5]

Percentage of users among the screened positive disabled, and total study population in square brackets (right bloc).

Ten persons with DD I were assigned no services

*ISSP* Individual support services plan
